# Copy Number Variation of *GSTT1* and *GSTM1* and the Risk of Prostate Cancer in a Caribbean Population of African Descent

**DOI:** 10.1371/journal.pone.0107275

**Published:** 2014-09-08

**Authors:** Elise Emeville, Cédric Broquère, Laurent Brureau, Séverine Ferdinand, Pascal Blanchet, Luc Multigner, Marc Romana

**Affiliations:** 1 Institut National de la Santé et de la Recherche Médicale (INSERM) U1085, Institut de Recherche sur la Santé, l’Environnement et le Travail (IRSET), Pointe-à-Pitre, Guadeloupe, France; 2 Université de Rennes 1, Rennes, France; 3 Institut National de la Santé et de la Recherche Médicale (INSERM) U1134, Pointe-à-Pitre, Guadeloupe, France; 4 Université des Antilles et de la Guyane, Pointe-à-Pitre, Guadeloupe, France; 5 Service d’Urologie, Centre Hospitalier Universitaire de Pointe à Pitre, Pointe à Pitre, Guadeloupe, France; National Cancer Institute, National Institutes of Health, United States of America

## Abstract

**Background:**

Deletions of the glutathione S-transferase genes M1 and T1 (*GSTM1* and *GSTT1*) have been studied as potential risk factors for prostate cancer. Conflicting results have been obtained. Moreover, most such studies could not discriminate heterozygous from homozygous carriers of the non-deleted alleles.

**Objective:**

We investigated whether copy number variation (CNV) of the *GSTM1* and/or *GSTT1* genes contribute to the risk of prostate cancer in the Caribbean population of African descent of Guadeloupe.

**Methods:**

In a population-based case-control study, we compared 629 prostate cancer patients and 622 control subjects. Logistic regression was used to estimate adjusted odds ratios (OR) and 95% confidence intervals (CI). Exact copy numbers of *GSTM1* and *GSTT1* were determined by real-time PCR.

**Results:**

A higher copy number of *GSTM1* was marginally associated with prostate cancer risk. Men with 2 and 3 or more *GSTT1* genes were at higher risk of prostate cancer (OR: 1.55, 95% CI: 1.11–2.16 and OR: 4.89, 95% CI: 1.71–13.99, respectively; P_trend_<0.001). Men with 3, 4 and 5 or more copies of both *GSTM1* and *GSTT1* genes were at higher risk of prostate cancer (OR: 2.18, 95% CI: 1.21–3.91, OR: 3.24, 95% CI: 1.63–6.46, and OR: 5.77, 95% CI: 1.40–23.84, respectively; P_trend_<0.001).

**Conclusions:**

Copy number of *GSTT1* and combined *GSTM1*/*GSTT1* appear to be associated with prostate cancer risk in our population study with gene dose relationship. Our results support the hypothesis that variations in copy number of *GSTT1* modulate the risk of prostate cancer.

## Introduction

Prostate cancer is one of the most commonly diagnosed malignancies in men [1]. It is disproportionately common among individuals of African descent, irrespective of the place where they live in the world, and less common in Caucasian and Asian populations [2]. The reasons for these ethnic differences in incidence are largely unknown but probably involve a complex interplay between hormonal, environmental and genetic factors [2], [3].

Genetic polymorphism of metabolic enzymes has been studied to investigate the possible etiological role of carcinogenic agents in prostate cancer [4]. Promising candidates include the glutathione S-transferase (GSTs) family, a group of phase II detoxifying enzymes that catalyze reactions between cytosolic glutathione and electrophilic substrates, producing stable and more soluble compounds that can then be excreted or compartmentalized [5].

It was assumed that GST-mediated conjugation resulted in less toxic or inactive metabolites. For two GST genes, *GSTM1* (GenBank: BC024005.2) and *GSTT1* (GenBank: BC007065.1), the complete deletion of the gene eliminates the gene function, leading to the inability to eliminate electrophilic compounds as efficiently; this may potentiate the deleterious effects of various environmental and endogenous carcinogens [6]. The main role of GSTs is the conjugation of reactive metabolites, but they may also be involved in producing reactive derivatives from the metabolism of halogenated compounds such as haloalkanes [7],[8]. Thus, the final direction of the effect - protection or susceptibility - on carcinogenesis, if any, is difficult to predict, and exposure to particular chemicals may alter the effect of GSTs on cancer risk.

Extensive research has been carried out, mostly in Caucasian and Asian populations, studying the relationship between *GSTM1* and *GSTT1* polymorphisms and prostate cancer risk. However, the results obtained are conflicting, and the putative associations identified remain a matter of debate [9]–[13]. A recent multi-institutional case-control study including 10 studies (1715 cases and 2363 controls) among subjects of African descent (African-American, African-Caribbean and African) has provided new data for this ethnic group [14]: homozygous deletion of *GSTM1* or of *GSTT1* was found to be inversely associated with the risk of prostate cancer. This suggests a paradoxical inverse effect of the loss of GST function on the risk of prostate cancer in these populations.

A major limitation of these studies is that they could not discriminate between heterozygous and homozygous carriers of the non-deleted alleles. Classifying such genotypes as carrier or non-carrier implies a recessive model (one or two copies versus the absence of the risk allele), which may not reflect the true underlying genetic mechanisms involved and thus may not provide a valid or accurate estimate of the genetic risk [15]. Moreover, the *GSTM1* and *GSTT1* genes exhibit copy number variation (CNV), and a dose effect between gene copy number and enzymatic activity has been reported for both genes [16]–[19]. As a consequence, analyses based on gene dose are likely to provide a better description of any association with disease outcome [20].

We recently described a population-based case-control study in Guadeloupe among a Caribbean population of African descent showing that homozygous deletions of *GSTM1* and those of *GSTT1* are each, independently, significantly associated with a reduced risk of prostate cancer [14]. Here, we report the continuation of this case-control study by determining the exact gene copy numbers of *GSTM1* and *GSTT1* genes and investigating the associations between the CNV of each gene and the risk of prostate cancer.

## Materials and Methods

### Ethics Statement

The study was approved by the Guadeloupe Ethics Committee for studies involving human subjects. Each participant provided written informed consent.

### Population Study

This study took place in Guadeloupe (French West Indies), a Caribbean archipelago, most of the inhabitants of which are of African descent. This study was carried out on 638 consecutive incident cases of histologically confirmed prostate cancer, and 628 controls without prostate cancer. Details of the selection of cases and controls have been described elsewhere [21]. Briefly, cases were recruited among patients at public and private urology clinics with a recruitment area covering the entire territory of the Guadeloupe Archipelago. Controls were recruited from men participating in a free systematic health screening program open to the general population: each year, a random population sample selected in accordance with the sex and age distribution of the general population was invited to participate; consecutive men aged 45 or older were invited to participate in this study, with selection according to the approximate age distribution of prostate cancer incidence in Guadeloupe. Inclusion criteria for both cases and controls were current residence in Guadeloupe, both parents born on any Caribbean island with a population of predominantly African descent, and no use of any drugs known to influence the hypothalamic-pituitary-gonadal-adrenal axis. Additional inclusion criteria for controls were normal findings upon digital rectal examination and a total plasma PSA concentration no higher than the 75th percentile for the corresponding age group of African American men without clinical evidence of prostate cancer [22]. All subjects were interviewed in person to obtain information about their age, Caribbean origin, education, weight and height allowing the calculation of body mass index (BMI, kg/m2), smoking, alcohol consumption, and history of prostate cancer screening within the last 5 years. Participants provided a blood sample.

### GSTM1 and GSTT1 copy number analysis

Genomic DNA was extracted from peripheral blood leukocytes by standard procedures and DNA was quantified using NanoVue Plus (GE Healthcare Bio-Sciences, Uppsala, Sweden). *GSTM1* and *GSTT1* copy number was determined using Taqman Gene Copy Number Assay designed by Applied Biosystems (Foster City, CA, USA). Briefly, dual real-time PCR were run on an Applied Biosystems StepOne Plus real-time PCR apparatus with gene-specific primers, a gene-specific 6-carboxyfluoresceine minor groove binder (6-FAM-MGB)-labeled probe (Hs01731033_cn and Hs02595872_cn for *GSTT1* and *GSTM1*, respectively), primers specific for the RNase P gene and a VIC-TAMRA probe for reference (4403326) (Table S1). Each target assay was run in the same PCR as RNase P. Genotyping was carried out blind to the case/control status of the subject. Samples were run in triplicate using 50 ng of genomic DNA. Quality control (QC) samples (water, blinded and not blinded samples) were included in genotyping assays. DNA samples containing 0 to 2 copies of the GSTM1 and GSTT1 genes previously assessed in the Taqman Gene Copy Number Assay were included on each genotyping plate, as internal quality controls. We used CopyCaller software v1 (Applied Biosystems) to quantify the gene copy number in each sample. A subsample of 20% of the samples was genotyped twice. The concordances for QC samples were 98% for both *GSTM1* and *GSTT1*. Discordant genotypes (n = 5) were excluded. Moreover, all subjects with more than two copies of *GSTM1* or *GSTT1* were systematically genotyped twice and the concordances were 100% for both genes. For 10 subjects, we failed to genotype GSTs.

### Statistical Analysis

The odds ratio (OR) and 95% confidence intervals (CIs) for the association between CNV of *GSTT1* and *GSTM1* genes and prostate cancer were estimated using unconditional logistic regression. Genotypes were coded as categorical variables (0, 1, 2, 3 or more gene copies for individual *GSTT1* or *GSTM1* analysis; 0, 1, 2, 3, 4, 5 or more copies for the sum of *GSTM1* and *GSTT1* genes). We investigated whether covariates were confounding factors by looking at the association between covariates and subject status (Table 1), as well as that between covariates and exposure according to the carrier status for *GSTM1*, *GSTT1* and the combined *GSTM1*/*GSTT1* copy numbers (Tables S2, S3 and S4 respectively). Confounding covariates included in the logistic model if they were associated (P<0.20) with both subject status and exposure. Age was always included in the adjusted model. Because log linearity of age was not achieved, age was categorized as quartiles according to the age distribution of the controls. Tests for trends in risk were performed by entering the categorical variable into the model as an ordinal variable. Missing data for covariates varied from none to 3 (0.2%) for PSA screening history, 6 (0.5%) for smoking, 12 (1.0%) for alcohol, and 34 (2.7%) for education. Missing data were handled by substituting them by a missing value indicator variable. We also considered possible interactions between CNV of *GSTT1* and *GSTM1* genes and selected covariates (smoking, alcohol consumption, BMI, family history of prostate cancer) in relation to the risk of prostate cancer. The P value for interaction was calculated by the likelihood ratio test comparing the log-likelihood for the model with the interaction terms to the log-likelihood for the model without the interaction term. SAS software version 9.3 (SAS Institute, Cary, NC) was used for all statistical analyses. All tests were two-tailed, and P values less than 0.05 were considered significant.

**Table 1:** Baseline characteristics of the study population.

Characteristics	Casesn = 629	Controlsn = 622	P-values^a^
**Age** (mean, range)	66.1 (45.8–94.5)	60.9 (45.1–88.8)	<0.001
**Caribbean origin** (n, %)			
French West Indies	609 (96.8)	572 (92.0)	<0.001
Haiti or Dominica	20 (3.1)	50 (8.0)	
**Education** (n, %)			
Primary	384 (61.4)	342 (57.8)	0.02
Secondary	156 (25.0)	187 (31.6)	
High school and higher	85 (13.6)	63 (10.6)	
**Body mass index** (kg/m^2^) (n, %)			
<25	295 (46.9)	293 (47.1)	0.53
25–<30	266 (42.3)	250 (40.2)	
≥30	68 (10.8)	79 (12.7)	
**Smoking** (n, %)			
Never	388 (62.3)	383 (61.9)	0.88
Former or current	235 (37.7)	236 (38.1)	
**Alcohol consumption** (n, %)			
Never	84 (13.5)	97 (15.9)	0.24
Former or current	537 (86.5)	514 (84.1)	
**PSA screening history** (n, %) b			
No	300 (47.9)	536 (86.2)	<0.001
Yes	326 (52.1)	86 (13.8)	
**Family history of prostate cancer**(n, %)			
No	346 (55.0)	478 (76.8)	<0.001
Yes	148 (23.5)	63 (10.1)	
Not known	135 (21.5)	81 (13.1)	
***GSTM1*** ** copy number** (n, %)			
0	159 (25.3)	197 (31.7)	0.02
1	317 (50.4)	284 (45.7)	
2	142 (22.5)	137 (22.0)	
3	8 (1.3)	4 (0.6)	
4	3 (0.5)	0 (0)	
***GSTT1*** ** copy number** (n, %)			
0	153 (24.3)	192 (30.9)	<0.001
1	280 (44.5)	284 (45.7)	
2	173 (27.5)	141 (22.7)	
3	15 (2.4)	3 (0.5)	
4	8 (1.3)	2 (0.3)	

a *P* values for continuous variables are those for non-parametric Mann-Whitney rank tests; for categorical variables, *P* values were calculated in tests for heterogeneity across levels.

## Results

The general characteristics of the study participants and the frequencies of the numbers of gene copies of *GSTM1* and *GSTT1* among the 629 cases and 622 controls are summarized in Table 1.

The frequency of homozygous deletion (0 copies) of *GSTM1* gene in the control group was 0.32 (95% CI, 0.28–0.35) and that of *GSTT1* was 0.31 (95% CI, 0.27–0.34). The frequency of homozygous deletions at both loci in the control group was 0.07 (95% CI, 0.05–0.09).

Crude and adjusted ORs for prostate cancer according to the copy number of *GSTM1* and *GSTT1* genes are given in Table 2. According to the adjusted model *GSTM1* carriers were at higher risk of prostate cancer (OR: 1.31, 95% CI: 1.01–1.71) than men with the homozygous *GSTM1* deletion. Men with at least 3 *GSTM1* genes were at a higher, but not significantly higher, risk of prostate cancer (OR: 2.55, 95% CI: 0.78–8.39); the trend for *GSTM1* gene dose association was inconclusive (P_trend_ 0.17). According to adjusted models, *GSTT1* carriers were at a higher risk of prostate cancer (OR: 1.40, 95% CI: 1.07–1.83) than men with the homozygous *GSTT1* deletion. Men with at least 2 and those with 3 or more *GSTT1* genes were at significantly increased risk of prostate cancer (OR: 1.55, 95% CI: 1.11–2.16 and OR: 4.89, 95% CI: 1.71–13.99, respectively), and there was a significant gene dose relationship of the *GSTT1* gene (P_trend_<0.001). Using subjects with homozygous deletion of both *GSTM1* and *GSTT1* genes as the reference group, adjusted models indicated that subjects carrying both genes were at significantly higher risk of prostate cancer (OR: 1.88, 95% CI: 1.11–3.21). Finally, men with 3, 4, and 5 or more copies of *GSTM1* and *GSTT1* genes were at significantly increased risk of prostate cancer (OR: 2.18, 95% CI: 1.21–3.91, OR: 3.24, 95% CI, 1.63–6.46, and OR: 5.77, 96% CI: 1.40–23.8, respectively) and there was a significant gene dose relationship (P_trend_<0.001).

**Table 2:** Association between copy number of the *GSTM1* and *GSTT1* genes and risk of prostate cancer.

	Cases	Controls	Crude OR (95% CI)	Adjusted OR (95% CI)^a^
***GSTM1*** ** copy number**	**n = 629 (%)**	**n = 622 (%)**		
0	159 (25.3)	197 (31.7)	1.0	1.0
1	317 (50.4)	284 (45.7)	1.38 (1.06–1.80)	1.37 (1.04–1.82)
2	142 (22.6)	137 (22.0)	1.28 (0.94–1.76)	1.28 (0.84–1.62)
≥3	11 (1.7)	4 (0.6)	3.40 (1.06–10.9)	2.55 (0.78–8.39)
*P*_trend_			0.03	0.17
Non-Carrier	159 (25.3)	197 (31.7)	1.0	1.0
Carrier	470 (74.7)	425 (68.3)	1.09 (0.78–1.51)	1.31 (1.01–1.71)
***GSTT1*** ** copy number**				
0	153 (24.3)	192 (30.9)	1.0	1.0
1	280 (44.5)	284 (45.7)	1.24 (0.95–1.62)	1.26 (0.94–1.68)
2	173 (27.5)	141 (22.7)	1.54 (1.13–2.09)	1.55 (1.11–2.16)
≥3	23 (3.7)	5 (0.8)	5.77 (2.14–15.5)	4.89 (1.71–13.99)
*P*_trend_			0.0002	0.0006
Non-Carrier	153 (24.3)	192 (30.9)	1.0	1.0
Carrier	476 (75.7)	430 (69.1)	1.38 (1.08–1.77)	1.40 (1.07–1.83)
**Sum of ** ***GSTM1*** **/** ***GSTT1*** ** copy numbers**				
0^b^	33 (5.2)	45 (7.2)	1.0	1.0
1^c^	160 (25.4)	188 (30.3)	1.16 (0.71–1.91)	1.56 (0.88–2.76)
2^d^	219 (34.9)	236 (37.9)	1.26 (0.78–2.06)	1.71 (0.98–2.99)
3^e^	143 (22.7)	114 (18.3)	1.71 (1.02–2.85)	2.18 (1.21–3.91)
4^f^	61 (9.7)	36 (5.8)	2.31 (1.25–4.25)	3.24 (1.63–6.46)
≥5^g^	13 (2.1)	3 (0.5)	5.90 (1.55–22.4)	5.77 (1.40–23.8)
*P*_trend_			<0.0001	<0.0001
Non-Carrier	33 (5.2)	45 (7.2)	1.0	1.0
Carrier	596 (94.8)	577 (92.8)	1.41 (0.89–2.23)	1.88 (1.11–3.21)

a Unconditional logistic regression adjusted for age and education for *GSTM1*; for age and family history of prostate cancer for *GSTT1*; for age and PSA screening history for the sum of *GSTM1* and *GSTT1* alleles.

b *GSTM1*_0_ and *GSTT1*_0_;

c *GSTM1*_0_ and *GSTT1*_1_ or *GSTM1*_1_ and *GSTT1*_0_;

d *GSTM1*_1_ and *GSTT1*_1_ or *GSTM1*_0_ and *GSTT1*_2_ or *GSTM1*_2_ and *GSTT1*_0_;

e *GSTM1*_2_ and *GSTT1*_1_ or *GSTM1*_1_ and *GSTT1*_2_ or *GSTM1*_3_ and *GSTT1*_0_ or *GSTM1*_0_ and *GSTT1*_3_;

f *GSTM1*_2_ and *GSTT1*_2_ or *GSTM1*_3_ and *GSTT1*_1_ or *GSTM1*_1_ and *GSTT1*_3_ or *GSTM1*_4_ and *GSTT1*_0_ or *GSTM1*_0_ and *GSTT1*_4_;

g *GSTM1*_3_ and *GSTT1*_2_ or *GSTM1*_2_ and *GSTT1*_3_ or *GSTM1*_4_ and *GSTT1*_3_ or *GSTM1*_4_ and *GSTT1*_1_ or *GSTM1*_1_ and *GSTT1*_4_ or *GSTM1*_4_ and *GSTT1*_2_.

An analysis of the interaction between smoking, alcohol consumption, body mass index, family history of prostate cancer and *GSTM1*, *GSTT1*, and the sum of *GSTM1*/*GSTT1* copy numbers is shown in Tables 3 to 5. The interaction between *GSTT1* copy number and smoking status resulted in a tendency towards a higher risk of prostate cancer risk only in individuals who had never smoked (Table 4). By contrast, the interaction between *GSTM1* or the sum of *GSTM1*/*GSTT1* copy numbers and alcohol consumption resulted in a tendency towards an increase in the risk of prostate cancer restricted to former or current drinkers (Tables 3 and 5). However, these interactions were not significant.

**Table 3:** Association between copy number of the *GSTM1* gene and risk of prostate cancer according to smoking, alcohol consumption, body mass index and family history of prostate cancer.

	GSTM1 Copy Number						P_trend_	P_interaction_
	0		1		≥2			
	Cases/Controls	OR^a^ (CI95%)	Cases/Controls	OR^a^ (CI95%)	Cases/Controls	OR^a^ (CI95%)		
**Smoking**								
Never	98/114	1.0	200/173	1.11 (0.73–1.68)	90/96	1.36 (0.95–1.95)	0.58	0.71
Former or Current	61/80	1.0	113/103	1.31 (0.77–2.26)	61/53	1.26 (0.79–2.02)	0.30	
**Alcohol**								
Never	24/29	1.0	42/47	0.98 (0.40–2.38)	18/21	0.94 (0.45–1.95)	0.95	0.51
Former or Current	132/163	1.0	270/223	1.25 (0.88–1.79)	135/128	1.48 (1.09–2.02)	0.18	
**BMI**								
<25	75/88	1.0	157/145	1.23 (0.82–1.86)	63/60	1.16 (0.70–1.92	0.51	0.69
≥25	84/109	1.0	160/131	1.52 (1.03–2.24)	90/89	1.21 (0.79–1.87)	0.37	
**Family history of** **prostate cancer**								
Without	88/147	1.0	186/208	1.34 (0.94–1.91)	72/123	0.88 (0.58–1.34)	0.64	0.67
With	46/21	1.0	64/28	1.11 (0.53–2.30)	38/14	1.14 (0.48–2.68)	0.76	

a Adjusted for age and education.

**Table 4:** Association between copy number of the *GSTT1* gene and risk of prostate cancer according to smoking, alcohol consumption, body mass index and family history of prostate cancer.

	GSTT1 Copy Number						P_trend_	P_interaction_
	0		1		≥2			
	Cases/Controls	OR^a^ (CI95%)	Cases/Controls	OR^a^ (CI95%)	Cases/Controls	OR^a^ (CI95%)		
**Smoking**								
Never	89/125	1.0	177/176	1.53 (1.06–2.22)	122/82	2.06 (1.35–3.14)	0.0007	0.21
Former or Current	63/66	1.0	100/105	0.93 (0.57–1.51)	72/65	1.27 (0.75–2.16)	0.37	
**Alcohol**								
Never	20/28	1.0	31/48	1.00 (0.46–2.19)	33/21	2.31 (0.99–5.40)	0.05	0.36
Former or Current	132/161	1.0	244/228	1.28 (0.93–1.76)	161/125	1.60 (1.12–2.29)	0.01	
**BMI**								
<25	74/94	1.0	138/137	1.29 (0.84–1.96)	83/62	1.79 (1.09–2.95)	0.02	0.95
≥25	79/98	1.0	142/145	1.27 (0.85–1.91)	113/86	1.61 (1.03–2.50)	0.03	
**Family history of** **prostate cancer**								
Without	87/146	1.0^b^	162/224	1.24^b^ (0.87–1.77)	97/108	1.63^b^ (1.09–2.45)	0.02	0.90
With	29/18	1.0	65/26	1.40^b^ (0.64–3.03)	54/19	1.92^b^ (0.84–4.35)	0.12	

a Adjusted for age and family history of prostate cancer.

b Adjusted for age.

**Table 5:** Association between copy number of the sum of *GSTM1*/*GSTT1* genes and risk of prostate cancer according to smoking, alcohol consumption, body mass index and family history of prostate cancer.

	Sum of GSTM1/GSTT1 Copy Genes								P_trend_	P_interaction_
	0		1		2		≥3			
	Cases/Controls	OR (CI95%)	Cases/Controls	OR^a^ (CI95%)	Cases/Controls	OR^a^ (CI95%)	Cases/Controls	OR^a^ (CI95%)		
**Smoking**										
Never	20/24	1.0	96/123	1.18 (0.55, 2.52)	139/142	1.47 (0.70, 3.10)	133/94	2.10 (0.98, 4.46)	0.004	0.48
Former or Current	13/20	1.0	64/64	2.11 (0.88, 5.09)	76/93	1.77 (0.75, 4.20)	82/59	2.88 (1.20, 6.88)	0.04	
**Alcohol**										
Never	6/7	1.0	21/28	0.91 (0.23, 3.54)	26/39	0.73 (0.19, 2.77)	31/23	1.62 (0.42, 6.22)	0.27	0.57
Former or Current	26/36	1.0	138/158	1.73 (0.91, 3.29)	188/190	2.00 (1.06, 3.75)	185/130	2.72 (1.44, 5.16)	0.0007	
**BMI**										
<25	15/21	1.0	83/93	1.66 (0.72, 3.84)	105/116	1.55 (0.77, 3.13)	92/63	1.89 (0.94, 3.86)	0.11	0.89
≥25	18/24	1.0	77/95	1.45 (0.67, 3.15)	114/120	1.36 (0.31, 5.91)	125/90	2.08 (0.47, 9.17)	0.06	
**Family history of prostate** **cancer**										
Without	20/34	1.0	92/142	1.40 (0.69, 2.87)	128/181	1.55 (0.77, 3.13)	106/121	1.89 (0.94, 3.86)	0.11	0.89
With	10/4	1.0	32/19	1.04 (0.23, 4.64)	49/24	1.36 (0.31, 5.91)	57/16	2.08 (0.47, 9.17)	0.06	

a Adjusted to age and PSA screening history.

## Discussion

To the best of our knowledge, this is the first study reporting the contribution of exact copy number variation of *GSTM1* and *GSTT1* genes to prostate cancer susceptibility in a population of African descent.

Our results indicate: first, that higher copy number of *GSTM1* tends to be associated, although not significantly, with an increased risk of prostate cancer; and second, that higher copy number of *GSTT1* is significantly associated with an increased risk of the disease. Furthermore, a higher combined *GSTM1* and *GSTT1* copy number appears to be significantly associated with an increased risk of prostate cancer.

We detected more than two copies of *GSTM1* or *GSTT1* genes in 3.4% of our study population: 1.2% for *GSTM1* and 2.2% for *GSTT1*. Evidence for duplication of *GSTM1* or *GSTT1* has been reported in Caucasian populations but at a substantially lower prevalence than in our study population. Among 10271 Danish subjects, only 24 individuals (0.2%) carried more than two copies of *GSTM1* or *GSTT1* [23]; in two studies of German subjects including 1320 [24] and 3602 [25] individuals, the frequency of *GSTM1* duplication was between 0.08% and 0%, and the frequency of *GSTT1* duplication 0% and 0.14%, respectively.

Previous association studies based on comparing carriers (irrespective of the copy number of the genes) and homozygous non-carriers of *GSTM1* and *GSTT1* genes with prostate cancer have given inconsistent results, leading to divergent conclusions [9]–[14]. There are several possible reasons for these discrepancies: differences in ethnic background, geographic origin, and/or in the environment of the populations studied; different definitions of control groups; and both small numbers of cases included and small effects of the genes leading to a lack of power. The most recent meta-analysis, mostly grouping studies conducted with Caucasian and Asian men, suggest that homozygous deletion of the *GSTM1* and *GSTT1* genes is associated with increased risk of prostate cancer [11]–[13]. Only one previous study assessed the relationship between the *GSTM1* and *GSTT1* copy numbers and prostate cancer [23]: it was a prospective study (The Copenhagen City Heart Study) of a Caucasian population of Danish descent and included 128 cases of prostate cancer. Lower *GSTT1* copy number was significantly associated with increasing cumulative incidence of prostate cancer and decreasing cumulative 5-year survival; no association was found with *GSTM1* copy number. Not only did our study not confirm these findings, but it even leads to the opposite conclusion for this population of African descent [14]: there was a positive association between *GSTM1* or *GSTT1* copy number and the risk of prostate cancer. These associations appear to be significant in among African-Caribbean and native African populations [14]. Our analysis of a Caribbean population of African descent suggests a relationship between the risk of prostate cancer and *GSTM1* gene copy number and shows a significant relationship between the risk and *GSTT1* gene copy number; consequently, it provides further evidence for the previously described inverse association between deletions of both GST genes and the risk of prostate cancer in men of African descent. These discrepancies between the findings of different studies remain unclear, but several explanations can be suggested.

The conjugation of glutathione prevent damage resulting from exposure to toxic chemicals and to normal oxidative products of cellular metabolism, the association between *GSTM1* and *GSTT1* copy number and increased risk of cancer is, at first glance, unexpected. However, GSTs and particularly *GSTT1* may also be involved in producing reactive derivatives with higher reactivity [7], [8]. Indeed, the increase in risk associated with higher GST gene copy number, for which a dose effect on the concentration of both enzymes has been shown [16]–[19], is consistent with the idea that functional GST alleles may result in the production of genotoxic metabolites from particular endogenous or environmental agents. It is entirely plausible that the men included in our study have been exposed to chemicals that are “activated” by *GSTM1* and/or *GSTT1* rather than “detoxified”. In such a scenario, carriers of large numbers of copies of the GST gene would generate and expose cells to higher concentrations of genotoxic products, thereby increasing the likelihood of carcinogenesis promotion. Alternatively, it is also possible that exogenous protective factors are substrates of GST. Were this to be the case, these compounds would be more rapidly eliminated through conjugation in *GSTM1*- and/or *GSTT1*-carriers, reducing the bioavailability of the putative anti-cancer compound.

Following stratification of the data for smoking status, the significant increase in prostate cancer risk associated with *GSTT1* copy number was found to be restricted to individuals who had never smoked. This finding is consistent with a previous observation of an association between homozygous *GSTT1* deletion and a lower risk of prostate cancer in Caribbean men who had never smoked [14]. Such observations contrast with those reported for African-American men, in whom homozygous *GSTM1* deletion was associated with an increase in the risk of prostate cancer among smokers, whereas homozygous *GSTT1* deletion was not [14]. The observed differences in such associations between Caribbean and African-American men suggest possible differences in various factors between these populations, including lifetime exposure to other carcinogens that saturate the GST system, different levels of tobacco consumption and possible differences in the composition of cigarettes between countries.

We found that current or former drinkers with more than two *GSTM1* or combined *GSTM1*/*GSTT1* gene copies had a greater risk of prostate cancer than teetotalers. No reports have yet been published concerning this genetic s × environment interaction and prostate cancer risk. Moreover, toxicological studies carried out *in vivo* and *in vitro* have reported conflicting results concerning the possible effects of alcohol on GST expression [26] and most epidemiologic studies have suggested that neither the amount nor the type of alcohol is clearly associated with a risk of developing prostate cancer [27], [28]. These observations should therefore be interpreted with caution until more data become available.

Guadeloupe is a Caribbean department of France characterized by the adoption of a Western lifestyle, including, in particular, eating habits that may be risk factors for prostate cancer [2], [3]. Moreover, since the middle of the 20th century, intensive banana farming in Guadeloupe led to the use of large amounts of chlordecone, an organochlorine insecticide, which has since been banned. This pesticide undergoes no significant biotic or abiotic degradation in the environment, so permanently polluted soils and waters remain a major source of contamination of foodstuffs, such that human beings continue to be exposed to this chemical [29]. Chlordecone is a potential carcinogen and has been associated with increased risk of prostate cancer in Guadeloupe [21]. Nevertheless, it is difficult to suggest that the exposure of our population to environmental agents is such that it can cause the association between prostate cancer and GSTs in the direction we observe.

We cannot exclude the possibility that our findings are due to the ethnic background of our population if a common inherited genetic trait is simultaneously associated with the disease and with a factor that may alters the balance between bioactivation and detoxification in the body. GST enzymes are involved in steroid metabolism, which is thought to be involved in the initiation or progression of prostate cancer [2], [3]. It has already been suggested that ethnic differences in the incidence of prostate cancer may be related to differences in lifelong androgen or estrogen exposure [2].

We are aware of the inherent limitations of patient-control studies. Several factors potentially generating bias must be considered, particularly those relating to differential errors in the measurement of disease or exposure. Patient identification was based on unequivocal histologic criteria and controls were selected on the basis of strict criteria, including normal findings on digital rectal examination and PSA in the normal range for age, taking the ethnic origin of the population into account. However, we cannot exclude the possibility that some control individuals had latent disease that was not detected by PSA analysis or digital rectal examination. However, undetected prostate cancer in control subjects would be expected to bias estimates toward the null hypothesis, so the positive association observed may be an underestimate. We recruited incident rather than prevalent patients, and controls were selected from a representative sample of the male Guadeloupean population during the study period. Differential misclassification of the GST genotypes with respect to case status is unlikely because the staff responsible for genotyping were blind to the case/control status of the subjects. Ethnic identification is always difficult and misclassification can never be excluded, particularly because mixed ancestry is very likely. At least 90% of the inhabitants of Guadeloupe are descended from slaves and immigrants from West and Central Africa. The remaining 10% of the population are descended from Indian immigrants during the XIX century, from more recent immigrants from the middle-east or Europeans. Our selection criteria, including only subjects whose parents were born in French West Indies or on any Caribbean island with a population of predominantly African descent (Haiti, Dominica), gave us some confidence in the homogeneity of our study population. Moreover, the frequency of homozygous deletions of *GSTM1* and *GSTT1* in our control population is consistent with that previously reported for Afro-American, Afro-Caribbean, Native African and Brazilian men of African descent [14], [30], [31]. Nevertheless, we cannot exclude the possibility that some unknown confounding factors remain that may account for the associations observed or that they are chance findings.

In summary, our study suggests that copy number of *GSTT1* and combined *GSTM1*/*GSTT1* copy number are associated to prostate cancer risk in men of African descent with gene dose relationship. Replication of these observations in other populations and mechanistic studies are needed before any causal link can be established.

## Supporting Information

Table S1 Information on TaqMan Copy Number Target Assay.
(DOC)

Table S2 Associations between *GSTM1* genotype and subject characteristics.
(DOC)

Table S3 Associations between *GSTT1* genotype and subject characteristics.
(DOC)

Table S4 Associations between combined *GSTM1* and *GSTT1* genotypes and subject characteristics.
(DOC)
