# Supplementary material for: Copy Number Variation of GSTT1 and GSTM1 and the Risk of Prostate Cancer in a Caribbean Population of African Descent
Source: PLoS One. 2014 Sep 8;9(9):e107275. doi: 10.1371/journal.pone.0107275 (PMC4157893; doi:10.1371/journal.pone.0107275)
Supplement: Table S1 — Information on TaqMan Copy Number Target Assay. (DOC) [file pone.0107275.s001.doc]

**Table S1: Information on TaqMan Copy Number Target Assay**

***GSTM1*:**

Applied Biosystem ® catalog number: Hs02595872_cn

Probe location: Chr.22:24376895 on NCBI build 37

Assay gene location: within exon 4

Amplicon length: 91 bp

***GSTT1*:**

Applied Biosystem ® catalog number: Hs01731033_cn

Probe location: Chr.1:110231936 on NCBI build 37

Assay gene location: overlaps exon 5 - intron 5

Amplicon length: 75 bp

***RNase P*:**

Applied Biosystem ® catalog number: 4403326

Probe location: chr.14:20811565 on NCBI build 37

Assay gene location: within the single exon of *RNase P* gene

Amplicon length: 87 bp
