# Supplementary material for: Copy Number Variation of GSTT1 and GSTM1 and the Risk of Prostate Cancer in a Caribbean Population of African Descent
Source: PLoS One. 2014 Sep 8;9(9):e107275. doi: 10.1371/journal.pone.0107275 (PMC4157893; doi:10.1371/journal.pone.0107275)
Supplement: Table S2 — Associations between GSTM1 genotype and subject characteristics. (DOC) [file pone.0107275.s002.doc]

**Table S2: Associations between *GSTM1* genotype and subject characteristics**

| **Characteristics** |  | **Non-carrier**  **(n = 356)** |  | **Carrier**  **(n = 895)** |  | ***P* ^a^** |
| --- | --- | --- | --- | --- | --- | --- |
|  |  |  |  |  |  |  |
| **Caribbean origin** (n, %) |  |  |  |  |  |  |
| French West Indies |  | 340 (95.5) |  | 841 (94.0) |  | 0.28 |
| Haiti or Dominica |  | 16 (4.5) |  | 54 (6.0) |  |
| **Education** (n, %) |  |  |  |  |  |  |
| Primary |  | 185 (54.6) |  | 541 (61.6) |  | 0.02 |
| Secondary |  | 100 (29.5) |  | 243 (27.7) |  |
| High school and higher |  | 54 (15.9) |  | 94 (10.7) |  |
| **Body mass index** (kg/m²) (n, %) |  |  |  |  |  |  |
| < 25 |  | 163 (45.8) |  | 425 (47.5) |  | 0.78 |
| 25 - < 30 |  | 148 (41.6) |  | 368 (41.1) |  |
| > 30 |  | 45 (12.6) |  | 102 (11.4) |  |
| **Smoking** (n, %) |  |  |  |  |  |  |
| Never |  | 212 (60.1) |  | 559 (62.9) |  | 0.35 |
| Former or current |  | 141 (39.9) |  | 330 (37.1) |  |
| **Alcohol consumption** (n, %) |  |  |  |  |  |  |
| Never |  | 53 (15.2) |  | 128 (14.5) |  | 0.74 |
| Former or current |  | 295 (84.8) |  | 756 (85.5) |  |
| **PSA screening history** (n, %) |  |  |  |  |  |  |
| No |  | 231 (65.1) |  | 605 (67.7) |  | 0.36 |
| Yes |  | 124 (34.9) |  | 288 (32.3) |  |
| **Family history of prostate cancer** (n, %) | | |  |  |  |  |
| No |  | 235 (67.1) |  | 589 (67.4) |  | 0.37 |
| Yes |  | 67 (19.2) |  | 144 (16.5) |  |
| Not known |  | 48 (13.7 |  | 141 (16.1) |  |

**^a^** *P* values from tests for heterogeneity across levels
