# Supplementary material for: Copy Number Variation of GSTT1 and GSTM1 and the Risk of Prostate Cancer in a Caribbean Population of African Descent
Source: PLoS One. 2014 Sep 8;9(9):e107275. doi: 10.1371/journal.pone.0107275 (PMC4157893; doi:10.1371/journal.pone.0107275)
Supplement: Table S3 — Associations between GSTT1 genotype and subject characteristics. (DOC) [file pone.0107275.s003.doc]

**Table S3: Associations between *GSTT1* genotype and subject characteristics**

| **Characteristics** |  | **Non-carrier**  **(n = 345)** |  | **Carrier**  **(n = 906)** |  | ***P* ^a^** |
| --- | --- | --- | --- | --- | --- | --- |
|  |  |  |  |  |  |  |
| **Caribbean origin** (n, %) |  |  |  |  |  |  |
| French West Indies |  | 323 (93.6) |  | 858 (94.7) |  | 0.46 |
| Haiti or Dominica |  | 22 (6.4) |  | 48 (5.3) |  |
| **Education** (n, %) |  |  |  |  |  |  |
| Primary |  | 193 (58.1) |  | 533 (60.2) |  | 0.80 |
| Secondary |  | 97 (29.2) |  | 246 (27.8) |  |
| High school and higher |  | 42 (12.6) |  | 106 (12.0) |  |
| **Body mass index** (kg/m²) (n, %) |  |  |  |  |  |  |
| < 25 |  | 168 (48.7) |  | 420 (46.4) |  | 0.05 |
| 25 - < 30 |  | 149 (43.2) |  | 367 (40.5) |  |
| > 30 |  | 28 (8.1) |  | 119 (13.1) |  |
| **Smoking** (n, %) |  |  |  |  |  |  |
| Never |  | 214 (62.4) |  | 557 (62.0) |  | 0.89 |
| Former or current |  | 129 (37.6) |  | 342 (38.0) |  |
| **Alcohol consumption** (n, %) |  |  |  |  |  |  |
| Never |  | 48 (14.1) |  | 133 (14.9) |  | 0.71 |
| Former or current |  | 293 (85.9) |  | 758 (85.1)   | |  | | --- | | | 758 | | --- | | 61.53 | | 72.12 | | 85.0 | | | --- | --- | --- | --- | --- | --- | --- |   85.0 |  |
| **PSA screening history** (n, %) |  |  |  |  |  |  |
| No |  | 239 (69.3) |  | 597 (66.1) |  | 0.29 |
| Yes |  | 106 (30.7) |  | 306 (33.9) |  |
| **Family history of prostate cancer** (n, %) | | |  |  |  |  |
| No |  | 233 (68.9) |  | 591 (66.7) |  | 0.13 |
| Yes |  | 47 (13.9) |  | 164 (18.5) |  |
| Not known |  | 58 (17.2) |  | 131 (14.8) |  |

**^a^** *P* values from tests for heterogeneity across levels
