# Supplementary material for: Copy Number Variation of GSTT1 and GSTM1 and the Risk of Prostate Cancer in a Caribbean Population of African Descent
Source: PLoS One. 2014 Sep 8;9(9):e107275. doi: 10.1371/journal.pone.0107275 (PMC4157893; doi:10.1371/journal.pone.0107275)
Supplement: Table S4 — Associations between combined GSTM1 and GSTT1 genotypes and subject characteristics. (DOC) [file pone.0107275.s004.doc]

**Table S4: Associations between combined *GSTM1* and *GSTT1* genotypes and subject characteristics**

| **Characteristics** |  | **Non-carrier**  **(n = 78)** |  | **Carrier**  **(n = 1173)** |  | ***P* ^a^** |
| --- | --- | --- | --- | --- | --- | --- |
|  |  |  |  |  |  |  |
| **Caribbean origin** (n, %) |  |  |  |  |  |  |
| French West Indies |  | 75 (96.2) |  | 1106 (94.3) |  | 0.49 |
| Haiti or Dominica |  | 3 (3.8) |  | 67 (5.7) |  |
| **Education** (n, %) |  |  |  |  |  |  |
| Primary |  | 41 (55.4) |  | 685 (59.9) |  | 0.69 |
| Secondary |  | 24 (32.4) |  | 319 (27.9) |  |
| High school and higher |  | 9 (12.2) |  | 139 (12.2) |  |
| **Body mass index** (kg/m²) (n, %) |  |  |  |  |  |  |
| < 25 |  | 36 (46.2) |  | 552 (47.1) |  | 0.25 |
| 25 - < 30 |  | 37 (47.4) |  | 479 (40.8) |  |
| > 30 |  | 5 (6.4) |  | 142 (12.1) |  |
| **Smoking** (n, %) |  |  |  |  |  |  |
| Never |  | 44 (57.1) |  | 727 (62.4) |  | 0.36 |
| Former or current |  | 33 (42.9) |  | 438 (37.6) |  |
| **Alcohol consumption** (n, %) |  |  |  |  |  |  |
| Never |  | 13 (17.3) |  | 168 (14.5) |  | 0.50 |
| Former or current |  | 62 (82.4) |  | 989 (85.5) |  |
| **PSA screening history** (n, %) |  |  |  |  |  |  |
| No |  | 45 (57.7) |  | 791 (67.6) |  | 0.07 |
| Yes |  | 33 (42.3) |  | 379 (32.4) |  |
| **Family history of prostate cancer** (n, %) | | |  |  |  |  |
| No |  | 54 (70.1) |  | 770 (67.1) |  | 0.64 |
| Yes |  | 14 (18.2) |  | 197 (17.2) |  |
| Not known |  | 9 (11.7) |  | 180 (15.7) |  |

**^a^** *P* values from tests for heterogeneity across levels
